# Mixed Signals: T Cells as Architects of IgE Immunity

**DOI:** 10.1111/imr.70119

**Published:** 2026-03-31

**Authors:** Abigail L. Tierney, Wallace P. Bezerra, Stephanie C. Eisenbarth, Adam Williams

**Affiliations:** ^1^ Department of Medicine, Division of Allergy and Immunology Northwestern University Feinberg School of Medicine Chicago Illinois USA; ^2^ Center for Human Immunobiology Northwestern University Feinberg School of Medicine Chicago Illinois USA

**Keywords:** IgE, IL‐13, IL‐4, iNKT, Tfh, Tfr, Th2, Treg

## Abstract

Food allergen‐specific IgE can cause significant pathology, yet the pathways that generate pathogenic, high‐affinity IgE remain incompletely understood. Increasing evidence suggests that IgE responses arise from the integration of multiple, and sometimes opposing, T cell–derived cues. T follicular helper (Tfh) cells shape affinity maturation within germinal centers and likely coordinate class switching, while tissue‐resident Th2 cells amplify local inflammation and potentially reinforce IgE production. Distinct Tfh subsets, characterized by differential production of IL‐4 and IL‐13, may further determine whether IgE responses remain low‐affinity or mature into high‐affinity antibodies capable of driving anaphylaxis. At the same time, regulatory populations—including regulatory T (Treg) cells and T follicular regulatory (Tfr) cells—impose restraints that can dampen or redirect humoral immunity. Early‐life antigen exposure may tip this balance toward durable tolerance or toward progressive diversification of pathogenic IgE. Together, these mixed signals from helper, regulatory, and innate‐like T cells likely determine the magnitude, affinity, and clinical impact of IgE responses. In this review, we explore how these intersecting pathways collectively regulate IgE immunity.

## 1 Introduction

Classical allergic disease (type I hypersensitivity) is defined by the presence of allergen‐specific IgE antibodies. Although IgE constitutes less than 0.01% of total serum immunoglobulin, even low concentrations are sufficient to trigger allergic symptoms and, in severe cases, life‐threatening anaphylaxis [1, 2, 3, 4, 5, 6]. IgE is induced in both helminth infection and allergy; however, helminths often elicit low‐affinity, polyclonal IgE, whereas allergic disease can be associated with high‐affinity IgE capable of driving robust mast cell degranulation [2, 4, 7, 8, 9, 10, 11, 12, 13, 14, 15]. T cell–derived IL‐4 is both necessary and sufficient for IgE class switching [16, 17, 18, 19, 20]. Although Th2 cells were initially described as the primary helpers of IgE production, T follicular helper (Tfh) cells are now recognized as the dominant source of IL‐4 within B cell follicles, consistent with their essential role in driving IgE responses [19, 21, 22, 23, 24, 25]. Affinity maturation classically occurs in germinal centers (GCs), yet IgE^+^ GC B cells are short‐lived, raising questions about how high‐affinity IgE is generated [12, 26, 27, 28]. Recent studies demonstrate that high‐affinity IgE can arise through sequential class switching from IgG1^+^ B cells [11, 15]. However, because sequential switching may also occur outside of secondary lymphoid organs (SLOs), non‐Tfh CD4^+^ T cell subsets may contribute to IgE generation in peripheral tissues [29, 30, 31, 32]. Below we discuss the diverse T cell subsets and cytokine signals that shape IgE responses in allergy and helminth immunity.

## 2 Paradigms of T Cell Help in IgE Production

As discussed below, our understanding of T cell help in IgE immunity continues to expand beyond classical Th2‐centric models to include specialized Tfh subsets that support IgE class switching and affinity maturation. In addition, it is becoming clear that regulatory and innate‐like T cell populations shape the magnitude, quality, and persistence of IgE responses to helminths and allergens.

### 2.1 Th2 Cells

A central function of CD4^+^ T cells is to provide help to B cells during humoral immune responses. Within the original Th1/Th2 paradigm, Th1 cells were associated with cellular immunity to intracellular pathogens and delayed‐type hypersensitivity reactions, whereas Th2 cells were viewed as the primary helpers of humoral immunity due to their production of IL‐4 [33]. In addition to promoting B cell survival, IL‐4 induces IgG1 and IgE class switching in vitro, and is essential for IgE production in vivo [16, 17, 18, 34, 35, 36] (See IL‐4 section below for more details). Early studies established IL‐4–producing Th2 cells as the canonical CD4^+^ T cell subset supporting humoral immunity, particularly IgE production. In retrospect, however, this model is fundamentally incomplete. First, while it was assumed that IL‐4‐producing Th2 cells localized to the anatomical sites of cognate T–B interactions—the B cell follicle or the GC—this was not clearly demonstrated in the literature. Instead, Th2 cells are now recognized to act primarily at peripheral tissue sites, with only a small fraction retained in SLOs [19, 23, 24, 25]. Second, in genetic models lacking Th2 differentiation (e.g., *Stat6* ^*−/−*^ or *Gata3* ^*−/−*^ mice), as well as under type 1–polarizing conditions such as viral infection that suppress Th2 responses, class‐switched antibody production other than IgE remains largely intact [37, 38, 39]. Collectively, these findings point to alternative CD4^+^ T cell subsets as the principal providers of effective B cell help, at least within SLOs.

### 2.2 Tfh Cells

For decades, Th2 cells were considered the canonical helper T cell subset responsible for coordinating humoral responses. This view was fundamentally revised with the discovery of Tfh cells—a distinct CD4^+^ T cell lineage uniquely specialized to support B cell differentiation and antibody production. The unique features of Tfh cells include their dependence on the transcription factor BCL6 and expression of the chemokine receptor CXCR5, which allow them to localize to the B cell follicle and/or the GC within SLOs [40, 41, 42]. Tfh cells are a key source of IL‐21 and CD40L and, by virtue of their localization within B cell follicles, are positioned to directly provide cytokine and costimulatory signals to cognate B cells during GC reactions, driving selection of high‐affinity, long‐lived antibodies. Soon after their initial discovery, several groups showed that a subset of Tfh cells—often termed Tfh2 cells—produce IL‐4 during helminth infection and in multiple allergy models [9, 19, 21, 22, 23, 24, 25, 43]. However, the relative contributions of IL‐4–producing Tfh cells versus Th2 cells to IgE antibody responses initially remained unclear.

During *Leishmania major* or *Heligmosomoides polygyrus bakeri* (*H. polygyrus*) infection, IL‐4–producing CD4^+^ T cells were found to localize within germinal centers of draining lymph nodes and to express canonical Tfh markers, including PD‐1, CXCR5, and BCL6 [21, 22]. These IL‐4^+^ Tfh cells formed conjugates with B cells undergoing class switching, providing direct in vivo evidence of Tfh‐mediated B cell help [21]. Consistent with this, IL‐4^+^ Tfh cells were enriched in mesenteric lymph nodes during *H. polygyrus* infection, whereas GATA3^+^ IL‐4^+^ Th2 cells predominated at peripheral sites [19]. Similar spatial segregation was observed in allergic airway inflammation, where *Il4*‐expressing CD4^+^ T cells in lung‐draining lymph nodes were largely CXCR5^+^ Tfh cells, while CXCR5^−^ *Il4‐*expressing cells accumulated in inflamed lung tissue [25]. Together, these studies demonstrate that although both Th2 and Tfh cells can express *Il4*, Tfh cells uniquely localize to B cell follicles and GCs, positioning them to provide effective help for B cell selection and IgE class switching.

Not only does their localization differ, but adoptive transfer experiments have shown that Tfh and Th2 cells can have distinct functions in vivo. Using a variety of inhaled allergen models, several groups have shown that transferred IL‐4‐producing Th2 cells drive allergic inflammation in the lung but are insufficient for IgE antibody production. One study showed that administration of ovalbumin (OVA) with the alarmin IL‐33 generated transcriptionally distinct *Il4*‐expressing CD4^+^ T cell populations: CXCR5^−^ ST2^+^ Th2 cells and CXCR5^+^ ST2^−^ Tfh cells [25]. Transfer of the CXCR5^−^ Th2 population into ILC‐deficient recipients followed by allergen challenge induced robust type 2 cytokine production and eosinophilic lung inflammation, demonstrating sufficiency for allergic airway inflammation. In contrast, transfer of these Th2 cells into T cell–deficient hosts failed to sustain antigen‐specific IgE responses, whereas transferred Tfh cells supported durable IgE production [25]. Using an inhaled peanut flour model, the same group found that adoptively transferred Th2 cells elicited only weak peanut‐specific IgE responses in T cell–deficient recipients, significantly lower than those supported by transferred Tfh cells [44]. The ability of Th2 cells to support IgE production was also examined during *Nippostrongylus brasiliensis* (*Nippo*) infection, where transfer of polyclonal Th2 cells into an irrelevant TCR transgenic host followed by re‐exposure to *Nippo* yielded anti‐*Nippo* IgE levels comparable to no‐transfer controls, whereas recipients of Tfh cells showed a trend toward increased parasite‐specific and total IgE, although this did not reach statistical significance [45]. Interpretation of these adoptive transfer studies is complicated by the marked plasticity of CD4^+^ T cells, as Th2 and Tfh cells can interconvert. Indeed, multiple studies have demonstrated that IL‐4–expressing PD‐1^−^ CXCR5^−^ Th2 cells can differentiate into IL‐4–producing Tfh cells following transfer [23, 45], whereas IL‐4–expressing PD‐1^+^ CXCR5^+^ Tfh cells can adopt a Th2 phenotype upon antigen re‐exposure in vivo [46]. Coupled with the inability of traditional genetic models to selectively ablate Th2 cells without affecting Tfh populations, this plasticity complicates assignment of Th2‐specific contributions to IgE responses within lymph nodes. Nonetheless, taken together, these experiments indicate that while Th2 cells are sufficient to drive allergic airway inflammation, they are largely insufficient to support robust, antigen‐specific IgE responses, in contrast to Tfh cells.

Using complementary genetic strategies to ablate or impair Tfh cells, numerous studies have demonstrated an essential requirement for Tfh cells in IgE production across helminth and allergy models. The most widely used approach is conditional deletion of the Tfh lineage–defining transcription factor BCL6 in T cells (*Cd4* ^*cre*^ *Bcl6* ^*fl/fl*^), which disrupts Tfh differentiation without eliminating Th2 cells [47]. Using this model, Meli et al. showed that Tfh‐deficient mice exhibit markedly reduced serum IgE and fewer IgE‐secreting cells in the mesenteric lymph node following *H. polygyrus* infection [19]. Notably, *Cd4* ^*cre*^ *Bcl6* ^*fl/fl*^ mice retained normal frequencies of GATA3^+^ IL‐4^+^ Th2 cells, yet still displayed impaired IgE responses, reinforcing the conclusion that Tfh—not Th2—cells provide the critical help required for IgE antibody production during type 2 immunity. *Cd4* ^*cre*^ *Bcl6* ^*fl/fl*^ Tfh‐deficient mice have also been used to interrogate IgE responses in food allergy models. In an oral peanut allergy model using cholera toxin as an adjuvant, we found that Tfh‐deficient mice failed to generate detectable peanut‐specific IgE after repeated sensitization, whereas floxed‐only controls mounted robust IgE responses [9, 48, 49]. These findings were independently confirmed by Xie and colleagues, who similarly reported an absence of total and peanut‐specific IgE in *Cd4* ^*cre*^ *Bcl6* ^*fl/fl*^ mice compared with *Bcl6* ^*fl/fl*^ controls [50]. Similar findings have been observed across multiple lung immunization models. For example, we have observed in an inhaled allergen model using *Alternaria alternata* (*Alternaria* hereafter) and NP‐conjugated OVA, Tfh‐deficient *Cd4* ^*cre*^ *Bcl6* ^*fl/fl*^ mice failed to generate NP‐specific IgE antibodies [9]. In addition, following inhalation of either *Alternaria* plus OVA or peanut flour alone, Tfh‐deficient *Cd4* ^*cre*^ *Bcl6* ^*fl/fl*^ mice failed to generate detectable OVA‐ or peanut‐specific IgE in the serum [25, 44]. Consistent with this, Tfh‐deficient mice in the inhaled peanut model were protected from systemic challenge, exhibiting no temperature drop or clinical signs of anaphylaxis, underscoring the essential role of Tfh cells in IgE production and allergic disease [25]. Using an alternative model of Tfh deficiency in which mice lack the IL‐6 receptor [51]—thereby disrupting STAT3‐dependent Tfh differentiation [41]—one study showed that sensitization with house dust mite and alum resulted in markedly reduced total serum IgE and negligible Der p1–specific IgE titers [51]. In contrast, we observe preserved Tfh cell numbers in T cell–specific STAT3‐deficient (*Cd4* ^*cre*^ *Stat3* ^*fl/fl*^) mice, and elevated IgE levels in a peanut–cholera toxin food allergy model [52]. Our findings are consistent with human disease, as patients with dominant‐negative IL‐6 receptor mutations or STAT3 loss‐of‐function mutations develop hyper‐IgE syndrome, despite STAT3‐deficient patients exhibiting reduced circulating Tfh cells compared with healthy controls [53, 54, 55, 56, 57].

Collectively, these findings suggest a refined paradigm in which Th2 and Tfh cells fulfill distinct, yet complementary roles defined by their anatomical localization. Th2 cells preferentially home to peripheral tissues, where they orchestrate type 2 inflammatory responses, whereas Tfh cells remain within SLOs to support GC reactions and mediate humoral immunity, including IgE class‐switch recombination (CSR). However, as is often the case in immunology, such a binary framework may be overly reductive. This model presupposes that CSR occurs exclusively within SLOs, yet multiple studies in both humans and mice have reported evidence of in situ IgE CSR within tissues [29, 30, 31, 32]. Moreover, recent data indicate that “IgE memory” is not maintained by IgE^+^ B cells themselves but rather by type 2–imprinted IgG1^+^ memory B cells (MBCII) that are predisposed to undergo secondary switching to IgE upon reactivation [11, 58, 59, 60]. There is also evidence of prior sequential class switching in IgE^+^ plasma cells from mice chronically exposed to allergen [61]. Together, these observations could support a revised model in which, during priming, Tfh cells generate both IgE B cells/plasma cells and a pool of MBCIIs that can subsequently undergo IgE switching within tissue sites under the influence of Th2 cells. The recently described proallergic Th2 (Th2a) and pathogenic effector Th2 (peTh2) populations, which are enriched in highly allergic individuals, may represent the effector T cells that mediate this tissue‐level reactivation [62, 63, 64, 65, 66]. Alternatively, local IgE CSR and affinity maturation could occur within tertiary lymphoid structures that develop within inflamed tissues, where functional Tfh cells have been documented. Dissecting the relative contributions of Tfh and Th2 cells to the overall IgE response remains a major experimental challenge. Although selective depletion of Tfh cells clearly abrogates IgE production, the absence of tools to selectively target Th2 cells has precluded definitive assessment of their independent contribution.

### 2.3 Regulatory T (Treg) Cells

Regulatory T cells (Tregs) are essential for maintaining tolerance to self‐antigens, commensal microbes, and dietary antigens thereby preventing autoimmunity, inflammation, and allergy [67, 68]. Tregs are defined by expression of the lineage‐defining transcription factor FOXP3 and high levels of the IL‐2 receptor α‐chain (CD25). They suppress immune activation through multiple mechanisms, including secretion of anti‐inflammatory cytokines such as IL‐10 and TGF‐β, expression of coinhibitory molecules like CTLA‐4, and consumption of IL‐2, limiting activation of IL‐2–dependent immune cells [67]. In both mice and humans, loss of Treg function due to mutations in FOXP3 results in profound immune dysregulation accompanied by early‐onset IgE‐mediated allergy [67, 69, 70]. In humans, such defects cause immune dysregulation polyendocrinopathy enteropathy X‐linked (IPEX) syndrome, most commonly due to mutations in the FOXP3 forkhead DNA‐binding domain [69, 71]. Interestingly, polymorphisms in non‐coding regions of the *FOXP3* gene have also been linked to allergy and autoimmunity in non‐IPEX patients [72]. Similarly, mice carrying the scurfy *Foxp3* mutation exhibit early, spontaneous elevations in serum IgE, accompanied by increased IL‐4 production, indicating unchecked type 2 immune skewing [70]. Together, these findings demonstrate that Tregs are essential for restraining type 2 cytokine production and preventing inappropriate IgE responses at steady state, motivating extensive investigation of Treg‐mediated control of IgE immunity in both mice and humans.

In humans, multiple correlative studies link reduced Treg frequencies with allergic disease and elevated allergen‐specific IgE [73, 74, 75]. In children with cow's milk allergy, Karlsson et al. reported lower frequencies of circulating CD4^+^CD25^+^ Tregs following milk challenge compared with children who had developed tolerance and outgrown their milk allergy [73]. Consistent with this, children with food allergy or atopic disease exhibit reduced frequencies of circulating CD4^+^CD25^+^FOXP3^+^ Tregs relative to healthy controls [74, 75]. Notably, in birch pollen allergy, peripheral Treg frequency is inversely correlated with serum allergen‐specific IgE levels [74]. Together, these observations support a role for Tregs in restraining allergen‐specific IgE antibody responses in humans.

The central role of regulatory T cells in immune tolerance is being therapeutically exploited in allergen‐specific immunotherapies (AIT), including oral (OIT), epicutaneous (EPIT), and sublingual (SLIT) immunotherapy, which aim to induce tolerance through repeated allergen exposure [76]. Numerous oral immunotherapy trials have demonstrated that desensitization to food antigens like peanut and egg is associated with an increase in antigen‐specific FOXP3^+^ Tregs [77] and FOXP3^−^ type 1 regulatory (Tr1) cells [78], reduced antigen‐specific serum IgE [78, 79, 80, 81], and increased IgG4 antibody titers [78, 79, 80, 81], resulting in a higher IgG4/IgE ratio—a hallmark of effective immunotherapy [82, 83, 84]. However, other studies report no changes in food‐specific Treg frequency or Treg transcriptional profiles, indicating that Treg induction does not uniformly correlate with sustained unresponsiveness across OIT cohorts [85, 86]. The mechanisms by which OIT‐induced regulatory populations enforce durable tolerance remain unclear and a major challenge for AIT is achieving sustained unresponsiveness after therapy cessation, potentially reflecting insufficient or unstable allergen‐specific Treg responses.

Complementing human studies, numerous mouse models have defined a central role for Tregs in suppressing IgE antibody production, particularly in the establishment of oral tolerance to food antigens. In the absence of adjuvant, oral antigen exposure typically induces tolerance rather than antigen‐specific IgE, a process associated with early induction of CD4^+^CD25^+^FOXP3^+^ Tregs [87]. Adoptive transfer of antigen‐specific Tregs is sufficient to suppress IgE responses following subsequent immunization with antigen plus adjuvant, underscoring their functional importance [75, 88]. Consistent with this, timed depletion of Tregs after antigen feeding but before systemic sensitization enhances antigen‐specific IgE production [89], while antibody‐mediated Treg depletion during peanut–cholera toxin sensitization similarly augments peanut‐specific IgE and type 2 cytokine production [90]. Together, these studies demonstrate that Tregs are critical regulators of oral tolerance and actively restrain type 2 immunity and IgE responses to food antigens. Our studies of hyper‐IgE–associated mutations further refine this model [52]. Although both DOCK8 and STAT3 deficiencies enhance Tfh2/13 differentiation (see IL‐13 section below) and are linked to hyper‐IgE syndromes, only DOCK8 deficiency is closely associated with food allergy. Accordingly, T cell–specific DOCK8‐deficient mice (*Cd4* ^*cre*^ *Dock8* ^*fl/fl*^) generate robust peanut‐specific IgE and are susceptible to anaphylaxis following unadjuvanted oral peanut exposure [52]. This is accompanied by increased numbers of Tfh13 cells and is coincident with defects in Treg and T follicular regulatory (Tfr) cells (see Tfr section below) populations. In contrast, T cell–specific STAT3‐deficient mice (*Cd4* ^*cre*^ *Stat3* ^*fl/fl*^) retain intact Treg and Tfr compartments and fail to generate IgE under tolerogenic conditions, despite enhanced Tfh2/13 differentiation [52]. However, acute ablation of Foxp3^+^ regulatory T cells in *Cd4* ^*cre*^ *Stat3* ^*fl/fl*^ *Foxp3* ^*DTR*^ mice unleashes pathogenic Tfh2/Tfh13 function and permits IgE induction. These findings support a two‐hit model in which DOCK8 deficiency simultaneously promotes pathogenic Tfh polarization and impairs regulatory T cell control, whereas intact Tregs in STAT3 deficiency serve as a critical gatekeeper preventing food allergen–specific IgE responses [52].

While the studies described above emphasize the suppressive role of classical FOXP3^+^ Tregs in allergic responses, more recent work has revealed substantial heterogeneity within the Treg compartment. At steady state and across multiple allergy models, subsets of Tregs can co‐express the Th2‐associated transcription factor GATA3 alongside FOXP3 [57, 75, 91, 92, 93, 94]. Under homeostatic conditions, GATA3^+^ Tregs appear to support tissue integrity, particularly in barrier sites such as the lung, gut, and skin [91, 92]. In contrast, several studies report a positive association between GATA3^+^ Tregs numbers and IgE production during allergic inflammation. For example, in food allergy–prone mice with hyperactive IL‐4 receptor signaling (*Il4ra* ^F709^), a subset of Tregs acquires a Th2‐like phenotype characterized by GATA3 and *Il4* expression [75]. Human studies have further suggested a positive link between Th2‐like Tregs and allergic disease. Tregs isolated from milk‐allergic children exhibit higher GATA3 expression than those from nonallergic controls, and upon in vitro stimulation with milk proteins, milk‐specific CD4^+^FOXP3^+^ cells from allergic—but not tolerant—donors can produce IL‐4 [75]. Similar findings have been reported in asthma, where CD4^+^CD25^+^ Tregs can express GATA3 and produce IL‐4 upon restimulation [95]. The emergence of Th2‐like Tregs correlates with increased total and antigen‐specific IgE levels and more severe food allergy, suggesting that Treg plasticity may contribute to allergic pathology under type 2–skewed conditions. Although these observations do not establish a causal role for GATA3^+^ Tregs in driving IgE responses, they raise the possibility that, in certain allergic contexts, Tregs may acquire effector‐like features or exhibit impaired suppressive function rather than enforcing tolerance. Supporting the latter interpretation, Noval Rivas and colleagues showed that in vitro–generated *Il4ra* ^F709^ Tregs failed to suppress IgE production and anaphylaxis upon transfer, unlike wild‐type Tregs [75]. However, Treg‐lineage–specific deletion of *Il4* and *Il13* ameliorated IgE responses and anaphylaxis, indicating that IL‐4 production by ’Th2 reprogrammed’ Tregs actively promoted allergic inflammation rather than enforcing tolerance. Whether wild‐type GATA3‐expressing Tregs actively promote IgE production or instead reflect defective regulatory function remains unresolved. Defining the role of effector‐like Treg programs will be critical for understanding how T cell regulation shapes IgE immunity.

Although extensive evidence underscores the central role of regulatory T cells in suppressing IgE production and enforcing tolerance, it remains unclear whether Tregs act directly on B cells to inhibit IgE class‐switch recombination or instead suppress upstream activation of antigen‐presenting cells and proinflammatory effector T cells. FOXP3^+^ Tregs, Tr1 cells, and LAP^+^ Tregs likely promote tolerance through multiple mechanisms, including secretion of IL‐10 and TGF‐β and expression of coinhibitory receptors such as CTLA‐4, PD‐1, and LAG‐3, thereby dampening dendritic cell and T cell activation [96]. In addition, in vitro co‐culture studies demonstrate that human Tregs can directly suppress IgE CSR and IgE secretion by B cells, indicating that direct regulation is also possible [97]. In vivo, however, conventional Tregs lack CXCR5 and localize to tissues or primarily to the T cell zone of SLOs, limiting their ability to directly interact with B cells in follicles or GCs [98]. Instead, Tregs likely restrain IgE responses indirectly by suppressing dendritic cell–T cell interactions and blocking Tfh differentiation [87, 99].

### 2.4 T Follicular Regulatory (Tfr) Cells

In recent years, Tfr cells have been identified as a distinct CD4^+^ T cell subset with features of both Tfh and Treg cells. In both mice and humans, Tfr cells co‐express BCL6 and FOXP3, localize to GCs, and express CD25 along with coinhibitory receptors such as CTLA‐4 [100, 101, 102, 103, 104]. Importantly, a genetic approach often used to deplete Tregs (e.g., *Foxp3* ^*cre*^ *‐DTR*) also depletes Tfr cells, complicating interpretation data using this model. Foundational studies in mice demonstrated that Tfr cells constrain GC magnitude by limiting both Tfh and GC B cell populations and by restricting GC output, including antigen‐specific memory B cells, plasma cells, and serum IgG levels [100, 101, 102, 105]. In the context of type 2 immunity, Tfr cells are generally thought to suppress IgE responses.

In human tonsils, a Tfr‐like population termed CD25^+^ follicular T (Tf) cells was shown to express CD25 together with high levels of PD‐1 and CXCR5 and produces IL‐10 upon restimulation, despite lacking FOXP3 expression [106]. Notably, frequencies of CD25^+^ IL‐10^+^ Tf cells, but not total Tregs or FOXP3^+^ Tf subsets, were inversely correlated with total serum IgE levels [106]. Functionally, CD25^+^ Tf cells exert suppressive effects on both T and B cells [106]. In vitro they repress total T cell and Tfh cell proliferation and dampen Tfh helper function by directly downregulating CD40L, IL‐21, and BCL6 expression by Tfh cells. CD25^+^ Tf cells also directly regulate B cell responses in vitro [106]. In Tfh–B cell co‐culture systems, CD25^+^ Tf cells reduced B cell proliferation, plasma cell differentiation, and IgE secretion [106]. Further, CD25^+^ Tf cells reduced ε germline transcript (εGLT, described further in the IL‐4 section of this review) production, an effect reversed by IL‐10 blockade, indicating that IL‐10 acts early to inhibit direct switching from IgM to IgE rather than sequential switching through IgG1. Although it remains unclear which B cell subsets are preferentially targeted in vivo, these findings suggest that human CD25^+^ Tf cells can suppress IgE responses by simultaneously restraining Tfh helper function and directly limiting B cell CSR.

Multiple studies in mice have highlighted a central role for Tfr cells in restraining IgE production at steady state and across diverse allergy models. One mechanism by which Tfr cells directly regulate GC responses involves neuritin, a neuropeptide highly expressed by Tfr cells that preferentially binds B cells [105]. Gonzalez‐Figueroa and colleagues showed that exogenous neuritin suppresses plasma cell differentiation in both mouse and human B cells in vitro [105]. In vivo, mice lacking neuritin specifically in *Foxp3*‐expressing cells developed spontaneous, tissue‐specific autoantibody responses and exhibited increased plasma cell frequencies following immunization, implicating neuritin as a key Tfr‐derived regulator of B cell responses [105]. Within the same study, genetic dissection of Tfr function using (*Foxp3* ^*cre*^ *Bcl6* ^*fl/fl*^) mice—which selectively ablates Tfr cells while preserving other FOXP3^+^ or BCL6^+^ populations [50, 105, 107, 108]—further supports this role [105]. In these mice, baseline serum IgE levels were elevated, and following immunization with either OVA plus alum or oral peanut plus cholera toxin, both total and antigen‐specific IgE responses were significantly increased compared with controls [105]. Together, these findings establish Tfr cells as critical suppressors of GC‐driven IgE responses and identify neuritin as an important mediator of Tfr–B cell regulation.

Additional studies have further defined the role of Tfr cells in restraining IgE responses using complementary in vitro and in vivo approaches [109]. In co‐culture experiments using Tfr, Tfh, and B cells isolated from house dust mite–exposed mice, inclusion of Tfr cells reduced the frequency of IgE^+^ B cells and IgE secretion relative to cultures containing only Tfh and B cells, demonstrating suppression of IgE class switching and antibody production [109]. Tfr cells also limited Tfh proliferation in vitro, and the remaining Tfh cells produced lower levels of type 2 cytokines, suggesting that Tfr cells can suppress Tfh13‐like responses [109]. To assess Tfr function in vivo, Clement and colleagues developed an inducible Tfr depletion model targeting FOXP3^+^CXCR5^+^ cells (*Foxp3* ^*cre*^ *Cxcr5* ^*fl‐STOP‐fl‐DTR*^) [109]. Depletion of Tfr cells following initial house dust mite exposure led to increased IgE^+^ plasma cells, elevated total and allergen‐specific serum IgE, and expansion of Tfh13 cells upon subsequent challenge [109]. Utilizing an NP‐OVA and CFA model, Clement and colleagues additionally reported that depletion of Tfr cells led to an increase in other serum antibody isotypes (IgG1, IgA), emphasizing that Tfr cells negatively regulate the GC response broadly rather than exclusively the IgE response [109]. These findings were recapitulated in a subsequent inhaled house dust mite aeroallergen model, in which mice lacking Tfr cells exhibited significantly increased total and allergen‐specific serum IgE compared with controls [94]. Together with described human tonsil studies, these findings support a model in which Tfr and Tfr‐like cells suppress IgE responses by negatively regulating Tfh cells and B cell differentiation during allergic immune responses.

In contrast to the predominantly suppressive role described above, two studies from the Dent lab have suggested that Tfr cells can promote IgE production in a peanut food allergy model [50, 108]. In this system, oral peanut plus cholera toxin sensitization of Tfr‐deficient mice (*Foxp3* ^*cre*^ *Bcl6* ^*fl/fl*^) resulted in loss of peanut‐specific IgE but a paradoxical increase in total serum IgE, accompanied by defects in Tfh and GC B cell populations [50]. Similar results were observed following depletion of all *Foxp3*‐expressing cells prior to peanut and cholera toxin sensitization, implying that Tfr cells may positively contribute to antigen‐specific IgE responses in this context [50]. To further explore this possibility, Xie and colleagues used a model with expanded Tfr populations (*Foxp3* ^*cre*^ *Pten* ^*fl/fl*^) and observed increased peanut‐specific and total IgE, as well as elevated IgG1 responses [50]. However, interpretation of these findings is complicated by concomitant increases in Tfh cells in *Foxp3* ^*cre*^ *Pten* ^*fl/fl*^ mice, which could independently enhance GC activity and IgE production. Notably, they found that Tfr cells were enriched for *Il10* expression and deletion of the IL‐10 receptor specifically on B cells resulted in reduced GC B cells and diminished peanut‐specific IgG1 and IgE following peanut and cholera toxin sensitization, implicating Tfr‐derived IL‐10 as a potential positive regulator of GC output and IgE responses [50]. Support for a role of Tfr cells in promoting GC responses also comes from work by the Craft lab, which showed that Tfr‐derived IL‐10 acts directly on GC B cells to enhance their differentiation and potentially affinity maturation during acute lymphocytic choriomeningitis virus (LCMV) infection [107]. The Dent lab also reported that Tfr cells express low levels of IL‐4 in their peanut‐cholera toxin food allergy model [108]. In Tfh–B cell co‐cultures, inclusion of IL‐4–sufficient Tfr cells increased the frequency of IgE^+^ B cells compared with IL‐4–deficient Tfr cells [108]. Consistently, in a food allergy model, adoptive transfer of IL‐4–deficient Tfr cells resulted in reduced antigen‐specific IgE relative to transfer of IL‐4–sufficient Tfr cells, whereas antigen‐specific IgG1 levels were comparable between groups [108]. Together, these data suggest that under specific immunization conditions, Tfr cells may promote IgE responses. Why these findings diverge from the broader literature remains unclear, but they underscore the context‐dependent nature of Tfr cell function in regulating GC responses and IgE immunity.

Collectively, most studies in humans and across diverse mouse allergy models support a central role for Tfr cells in limiting GC responses and restraining IgE production. However, the opposing findings described above highlight the importance of experimental context. Variations in the route of allergen exposure, the adjuvant used, or the timing and dose of antigen exposure may determine whether Tfr cells suppress or support IgE responses, even within the same genetic model. Such differences likely reshape the cytokine milieu within SLOs, shifting regulation toward IgE induction rather than suppression.

### 2.5 Natural Killer T (NKT) Cells

NKT cells are a subset of unconventional T cells whose T cell receptors (TCRs) recognize lipid antigens presented by CD1d [110]. In mice, a major subset, termed invariant NKT (iNKT) cells, expresses a semi‐invariant TCR composed of an invariant Vα14–Jα18 (also known as Vα14–Jα281) α chain paired with a limited set of β chains [111]. Mice lacking the Jα18 gene (*Jα18* ^*−/−*^, also referred to as *Jα281* ^*−/−*^) therefore lack iNKT cells [112]. Although numerous endogenous and microbial lipid antigens presented by CD1d have been identified, the glycolipid α‐galactosylceramide (α‐GalCer) is most commonly used to activate iNKT cells experimentally [111]. Upon activation, iNKT cells rapidly produce large amounts of cytokines, particularly IL‐4 and IFNγ, enabling them to shape downstream adaptive immune responses, including CD4^+^ T cell differentiation [111]. Consistent with their potent and early IL‐4 production, multiple studies have implicated iNKT cells in promoting IgE responses.

Across multiple mouse models of allergic airway inflammation, several groups have demonstrated that the absence of iNKT cells is associated with impaired antigen‐specific serum IgE responses and reduced production of the type 2 cytokines IL‐4, IL‐5, and IL‐13 in the lung [113, 114, 115, 116, 117]. In three of these studies, mice lacking CD1d or iNKT cells (*Jα18* ^*−/−*^) that were sensitized and subsequently challenged with OVA via the airway exhibited markedly reduced levels of OVA‐specific IgE in the serum compared with iNKT‐sufficient control mice [113, 115, 116, 117]. Notably, only one of these reports included co‐administration of a known iNKT cell agonist with OVA [113], whereas the remaining studies relied on alternative adjuvants, including alum [115, 116] or house dust extract [117]. These differences raise an important mechanistic question regarding how iNKT cells are activated in these models.

For example, in the house dust extract model, the authors hypothesized that some house dust preparations contain lipid antigens capable of activating iNKT cells in a manner analogous to α‐GalCer [117]. Consistent with this hypothesis, Wingender and colleagues demonstrated that select house dust extracts could activate iNKT cell lines in vitro [117]. Moreover, both adoptive transfer of house dust extract–loaded bone marrow–derived dendritic cells and direct immunization with OVA plus house dust extract resulted in iNKT cell activation in vivo [117]. Importantly, OVA‐specific IgE responses induced by OVA and house dust extract immunization were observed in wild‐type mice but were absent in iNKT‐deficient *Jα18* ^*−/−*^ mice, supporting a positive role for iNKT cells in promoting IgE production in this model [117]. However, the identity of the lipid antigen(s) within house dust extracts responsible for iNKT cell activation remains unknown.

In contrast, in two studies that reported impaired OVA‐specific IgE responses in Jα18^*−/−*^ mice following OVA and alum immunization, no exogenous lipid antigens such as α‐GalCer were present to directly activate iNKT cells [115, 116]. While identifying “natural antigens” capable of activating iNKT cells in a manner similar to synthetic α‐GalCer remains an active area of investigation, one prevailing hypothesis is that microbial products or endogenous self‐lipids activate iNKT cells [111].

Supporting the possibility of non–TCR‐dependent iNKT cell activation at later stages of allergic inflammation, Scanlon and colleagues reported that following an initial immunization with OVA plus the synthetic iNKT agonist PBS57, subsequent OVA‐only airway challenges led to substantial accumulation of iNKT cells in the lung despite the absence of exogenous lipid antigen [113]. Notably, this late‐stage accumulation was no longer dependent on CD1d, as in vivo blockade of CD1d had no effect on iNKT cell numbers in the lungs after bronchoalveolar lavage. These findings led the authors to propose that iNKT cells at this stage are activated through cytokine‐mediated, rather than TCR‐dependent, mechanisms.

Taken together, these studies suggest that in models lacking α‐GalCer or related agonists, iNKT cells may initially be activated indirectly through CD1d‐expressing antigen‐presenting cells presenting self‐lipids. With persistent antigen exposure, iNKT cells may subsequently undergo bystander activation driven by inflammatory cytokines rather than continued TCR engagement. This model provides a plausible framework for reconciling disparate experimental systems, though it remains to be tested directly.

Like the other T cell subsets discussed in this review, iNKT cells may promote IgE responses through either direct or indirect mechanisms. One prevailing model posits that iNKT cells act as an early source of polarizing cytokines during conventional CD4^+^ T cell priming, thereby shaping downstream humoral immunity. Evidence supporting this framework comes from studies using house dust extract or alum in combination with OVA [115, 116, 117]. Following airway challenge, cells in the bronchoalveolar lavage fluid from iNKT‐deficient *Jα18* ^*−/−*^ mice produced reduced IL‐4 compared with controls, consistent with impaired type 2 polarization [115, 116, 117]. When considered alongside the marked defects in OVA‐specific serum IgE observed in these animals, these findings support a model in which early iNKT cell activation after OVA plus adjuvant exposure (alum or α‐GalCer) leads to rapid IL‐4 production that conditions conventional CD4^+^ T cells, which subsequently engage B cells to promote IgE class‐switch recombination and antibody production.

This paradigm is further supported by results from a distinct allergic airway model in which mice were sensitized with ragweed in alum and later challenged intranasally with ragweed [114]. iNKT‐deficient mice exhibited reduced ragweed‐specific serum IgE following challenge, and lymph node cultures from sensitized *Jα18* ^*−/−*^ mice produced virtually no IL‐4 upon ragweed restimulation in vitro [114]. Conversely, administration of α‐GalCer to activate iNKT cells prior to ragweed exposure exacerbated ragweed‐specific IgE responses and enhanced IL‐4 production by splenocytes [114]. Together, these studies reinforce the concept that iNKT cell–derived IL‐4 amplifies type 2 CD4^+^ T cell responses that support IgE production. Although these reports predated the recognition of IL‐4–producing Tfh cells as the main CD4^+^ T cell subset responsible for supporting IgE class‐switch recombination, the data can now be reinterpreted through this lens. It is plausible that IL‐4^+^ Tfh cells are diminished in the draining lymph nodes of *Jα18* ^*−/−*^ mice in these models, thereby accounting for the reduced antigen‐specific IgE responses observed.

In contrast to these findings, Kojo and colleagues reported that following DNP–OVA and alum sensitization, repeated exposure to α‐GalCer resulted in reduced total and DNP‐specific serum IgE levels compared with mice that did not receive α‐GalCer [118]. Antigen‐specific IgG1 and IgG2c responses were similarly diminished, indicating a broad suppression of the humoral immune response following chronic iNKT cell activation. Notably, repeated α‐GalCer administration had no effect on serum IgE levels in iNKT‐deficient mice, demonstrating that this suppressive effect was iNKT cell–dependent. In a subsequent study, the same group showed that repeated α‐GalCer immunization attenuated dendritic cell activation in vivo in an iNKT cell–dependent manner, with dendritic cells from *Jα18* ^*−/−*^ mice resembling those from untreated mice [119]. Moreover, repeated α‐GalCer treatment enhanced IL‐10 production by dendritic cells upon ex vivo restimulation. When pulsed with OVA and co‐cultured with naïve OVA‐specific CD4^+^ T cells, dendritic cells isolated from mice repeatedly immunized with α‐GalCer were sufficient to induce a modest population of IL‐10–producing Tr1 cells in vitro [118]. Collectively, these data suggest that repeated iNKT cell activation by lipid antigens after sensitization can shift the immune response toward a more tolerogenic state, although the mechanisms underlying this transition in vivo remain incompletely defined. Notably, these studies place dendritic cells at the center of iNKT regulation of IgE responses. Indeed, iNKT cells are potent regulators of dendritic cell activation and function in both tissues and SLOs [120, 121, 122, 123]. Although direct experimental evidence is currently lacking, it is plausible that iNKT cells modulate dendritic function during allergic priming to promote the type 2–skewing environment that ultimately supports IgE induction.

iNKT cells could also directly promote IgE class switching through cognate interactions with B cells. In addition to producing IL‐4, iNKT cells express key costimulatory molecules such as CD40L that are required for B cell activation and IgE CSR. For iNKT cells to directly influence class switching, they must be appropriately positioned within SLOs to engage B cells. At steady state, iNKT cells predominantly reside in T cell zones; however, following immunization with α‐GalCer, a limited number can migrate into B cell follicles [124, 125]. Additionally, during influenza infection, iNKT cells have been shown to provide IL‐4 to non‐cognate B cells outside GCs, thereby supporting GC responses [126]. Together, these findings suggest that while iNKT cells can access follicular regions and deliver helper signals, the extent to which they directly engage B cells to regulate GC reactions remains unclear. More recently, several groups have demonstrated that a subset of iNKT cells can acquire a T follicular helper–like phenotype, termed “iNKTfh” cells [127, 128, 129]. These studies showed that iNKTfh cells upregulate CXCR5, enabling localization to B cell follicles and GCs, and provide direct help to CD1d‐expressing B cells via CD40L. In viral infection models and α‐GalCer–adjuvanted vaccination systems, iNKTfh cells were sufficient to support antibody responses [127, 128, 129]. Notably, Chang and colleagues demonstrated that iNKTfh cell differentiation is dependent on BCL6, underscoring the transcriptional parallels between conventional Tfh cells and CD1d‐restricted iNKTfh cells [129]. Although a specific role for iNKTfh cells in type 2 immunity or IgE responses has not yet been demonstrated, these findings raise the possibility that Tfh‐like iNKT cells could provide direct cognate help to CD1d‐expressing B cells in response to allergens or helminths following lipid antigen–mediated activation. Whether such interactions meaningfully contribute to IgE class switching in vivo remains an open question [130].

Together, these data suggest two non–mutually exclusive pathways by which iNKT cells may promote IgE production. First, upon activation by lipid antigens presented on CD1d during allergic priming, iNKT cells may rapidly produce IL‐4 in peripheral tissues and/or the draining lymph node. In tissues, iNKT‐derived IL‐4 could condition dendritic cells prior to their migration to secondary lymphoid organs, shaping their activation state and cytokine program. Once in the lymph node, this DC conditioning—together with IL‐4 produced locally by iNKT cells—could promote differentiation of naïve CD4^+^ T cells into IL‐4–producing Tfh cells that drive IgE class‐switch recombination. Second, iNKT cells that acquire a Tfh‐like phenotype (iNKTfh) may localize to B cell follicles and provide direct cognate help to CD1d‐expressing B cells through CD40L and IL‐4. Determining how iNKT‐derived IL‐4 shapes dendritic cell function in tissues versus lymph nodes, influences Tfh differentiation, and whether iNKTfh cells contribute to allergen‐ or helminth‐induced IgE responses remains an important area for future study.

## 3 T Cell Derived Cytokines That Regulate IgE

CD4^+^ T cell–derived cytokines shape B cell survival, proliferation, differentiation, and antibody class switching, thereby determining antibody effector function. These cytokines can act directly on B cells through cognate receptors or indirectly by modulating other CD4^+^ T cells or dendritic cells, ultimately influencing downstream B cell responses. Although cytokines are often categorized as either pro‐ or anti‐inflammatory, their functions are highly context dependent, and most can exert opposing effects depending on the cellular source, tissue environment, timing, and responding cell type. In type 2 immunity, IL‐4 is the central cytokine driving IgE class‐switch recombination and is both necessary and sufficient for this process. However, additional T cell–derived cytokines can further promote or restrain IgE antibody responses, as discussed below.

### 3.1 Interleukin‐4 (IL‐4)

IL‐4 was originally described as “B cell stimulatory factor‐1” or “B cell growth factor” following its discovery in 1982 as a T cell–derived cytokine distinct from IL‐1 and IL‐2 that promoted proliferation of activated murine B cells, switching to IgG1, and IgG1 secretion in vitro [34, 35]. In vitro, IL‐4 alone was sufficient to drive B cells into S phase at levels comparable to BCR stimulation, while combined BCR and IL‐4 signaling produced maximal proliferation [131]. Subsequent studies demonstrated that IL‐4 could induce IgE production in LPS‐activated mouse B cells in vitro [16, 18], findings later confirmed in human B cell cultures, where IL‐4 induced εGLTs and IgE secretion [17, 132]. Notably, among 16 cytokines tested by Gauchat and colleagues, IL‐4 was uniquely capable of inducing εGLTs and IgE class‐switch recombination in vitro [133]. The molecular mechanisms by which IL‐4 directly induces CSR are well defined. IL‐4 signaling activates STAT6, which binds the Iε promoter and recruits transcriptional regulators including E2A proteins, NFIL3, and NF‐κB. Together, these factors initiate transcription of εGLTs [134, 135, 136, 137, 138], an obligate prerequisite for CSR that opens the Cε locus and targets activation‐induced cytidine deaminase (AID) to the ε switch region.

Extensive evidence shows that IL‐4 is essential for IgE class switching in vivo, as IL‐4 blockade or deficiency abolishes serum IgE responses in helminth infection and allergen immunization models [19, 24, 36, 37, 108]. This central role for IL‐4 in type 2 immunity and IgE production has been successfully exploited therapeutically in allergic disease. Dupilumab, a human monoclonal antibody targeting the IL‐4 receptor α chain (IL‐4Rα) to block both IL‐4 and IL‐13 signaling, is approved for the treatment of asthma, atopic dermatitis, and eosinophilic esophagitis [139]. Yet disappointingly, although IL‐4Rα blockade reduces IgE levels in food allergy and modestly enhances the efficacy of peanut OIT in children and adolescents, it does not induce durable tolerance or protect against OIT‐related anaphylaxis [140, 141, 142, 143].

#### 3.1.1 Regulation of IL‐4 in T Cells

In T cells, *Il4* expression is controlled by multiple transcription factors. In murine Th2 cells, STAT6 and GATA3 are central drivers of *Il4* expression, operating through a positive feedback loop in which IL‐4–mediated STAT6 activation induces GATA3, which in turn promotes transcription at the type 2 cytokine locus [144]. Disruption of either STAT6 or GATA3 impairs IL‐4 production by mouse CD4^+^ T cells in vitro [38, 39, 145, 146] and results in markedly reduced IgE responses in vivo [38, 39, 147].

This molecular pathway was described prior to the identification of Tfh cells. Notably, GATA3 expression is actively repressed by BCL6 in both murine and human Tfh cells [148, 149], raising a fundamental question as to which transcription factor(s) drive *Il4* expression in the Tfh lineage. Despite this antagonistic relationship, several studies have reported co‐expression of BCL6 and GATA3 in Tfh cells [9, 23, 45, 150, 151]. However, this phenomenon appears to be highly model dependent, as multiple reports have concluded that GATA3 is not expressed in Tfh cells [24, 33, 34, 115, 116, 117], even though some of these same studies documented *Il4* expression within the Tfh compartment [24, 152, 153, 154].

One transcription factor implicated in promoting *Il4* expression in Tfh cells is BATF (basic leucine zipper ATF‐like transcription factor). Using an OVA and alum immunization model, Sahoo and colleagues demonstrated that BATF‐deficient mice exhibit a selective loss of *Il4* expression in Tfh cells, which was accompanied by a markedly blunted IgE antibody response [154]. Mechanistically, chromatin binding analyzes revealed that BATF directly associates with the conserved noncoding sequence 2 (CNS2) enhancer located 3′ of the *Il4* locus [155, 156, 157]. In Tfh cells, *Il4* expression is uniquely dependent on CNS2, whereas CNS2 is dispensable for *Il4* expression in Th2 cells, highlighting distinct regulatory mechanisms across helper T cell subsets [155, 156]. Extending this model, Sahoo and colleagues showed that IL‐4/IL‐4 receptor signaling through STAT6 induces BATF expression in Tfh cells, and that BATF, together with IRF4, binds CNS2 to drive *Il4* transcription [154]. Interestingly, they also observed STAT3 binding to CNS2 and showed that Tfh cells from STAT3‐deficient mice expressed little to no *Il4*, indicating a role for STAT3 in promoting *Il4* expression in Tfh cells. However, in a mouse model of food allergy, we recently showed that STAT3‐deficient Tfh cells expressed elevated IL‐4 levels, meaning STAT3‐mediated regulation of *Il4* expression in Tfh cells is likely context dependent [52].

These data indicate that *Il4* expression in Tfh cells can be driven by transcription factors other than GATA3. However, in models in which Tfh cells express GATA3, it remains unclear whether *Il4* expression is GATA3 dependent or whether other factors, such as BATF, are sufficient. Moreover, the signals that induce GATA3 expression in Tfh cells in some contexts but not others remain undefined, as well as how Tfh cells co‐express GATA3 and BCL6 despite their antagonistic relationship.

### 3.2 IL‐21

IL‐21 is a signature cytokine of Tfh cells [41, 45, 158] and signals predominantly through STAT3 [41, 45, 158]. In concert with IL‐4, IL‐21 broadly supports GC responses by promoting B cell survival and proliferation [159, 160], inducing and maintaining BCL6 expression in GC B cells [161, 162], and driving the generation of antigen‐specific serum antibodies [162, 163]. Therefore, Tfh‐derived IL‐4 and IL‐21 are considered to share substantial functional overlap in sustaining GC reactions in vivo.

Despite these shared roles, several studies suggest that IL‐21 performs functions distinct from IL‐4 during a humoral response. Notably, IL‐21 has been implicated in supporting the formation and maintenance of Tfh cells. Mice deficient in IL‐21 or the IL‐21 receptor exhibit impaired Tfh cell development in multiple experimental systems [41, 163, 164]. However, this requirement appears to be context dependent, as other studies using similar genetic models have reported minimal or no defects in Tfh cell populations [160, 162, 165]. Impaired IL‐21 signaling also directly affects B cell responses. Across multiple vaccination models, IL‐21– or IL‐21 receptor–deficient mice exhibit reduced total and antigen‐specific GC B cells, impaired antigen‐specific IgG1 production, and defective memory B cell formation^,124^. Consistent with these findings, humans with IL‐21 receptor deficiency show reduced frequencies of class‐switched B cells in peripheral blood, and naïve B cells from these patients display impaired proliferation and class switching in vitro compared with healthy controls [166]. Together, these data underscore the essential role of IL‐21 in supporting humoral immunity in both mice and humans.

IL‐21 signaling also shapes IgE responses, generally opposing IL‐4–driven IgE production. At steady state, IL‐21–deficient mice exhibit elevated serum IgE compared with wild‐type controls [162], a phenotype mirrored in patients with IL‐21 receptor loss‐of‐function mutations [166]. Consistent with this, across multiple protein vaccination models, mice lacking IL‐21 signaling (*Il21* ^*−/−*^ or *Il21r* ^*−/−*^) display increased frequencies of IgE^+^ germinal center B cells and antigen‐specific IgE^+^ plasma cells, as well as elevated total and/or antigen‐specific serum IgE [160, 163, 167]. Conversely, administration of exogenous IL‐21 during OVA immunization suppresses OVA‐specific IgE production in vivo [168]. In contrast, recent work suggests that IL‐21 promotes the generation of IgG1^+^ MBCIIs, proposed precursors of allergen‐specific IgE plasma cells [169], implying a potential indirect role for IL‐21 in supporting IgE responses. However, this interpretation warrants caution, as IL‐21–deficient mice in these models still exhibit increased IgE plasma cells. Overall, data from both mice and humans support a model in which IL‐21 broadly promotes humoral immunity while being associated with suppression of IgE responses in vivo.

In vitro culture systems using murine and human B cells have revealed unexpected complexity in how IL‐21 regulates IgE class switching and antibody production. In cultures of naïve mouse B cells stimulated with mitogens or CD40 ligation in the presence of IL‐4, multiple studies have shown that addition of IL‐21 reduces the frequency of IgE^+^ B cells or levels of secreted IgE compared with IL‐4 alone [138, 167, 168]. In contrast, studies using human B cells have reported both inhibitory and enhancing effects of IL‐21 on IgE production, depending on experimental conditions. Avery and colleagues demonstrated that IL‐21 cooperates with IL‐4 and multimeric CD40 ligand to increase IgE secretion relative to IL‐4 and CD40 co‐stimulation alone, an effect dependent on STAT3 signaling [170]. Consistent with this, B cells from patients with STAT3 loss‐of‐function mutations secreted significantly less IgE when cultured with IL‐4 and IL‐21 than did B cells from healthy controls, whereas no difference was observed in cultures containing IL‐4 alone. Additional studies similarly showed that IL‐21, in combination with IL‐4 and anti‐CD40 stimulation, enhances IgE secretion from naïve or memory human B cells compared with cultures lacking IL‐21 [171, 172], supporting a model in which IL‐21 can promote IgE production in vitro. Conversely, other reports suggest that IL‐21 inhibits IgE production by human B cells. Human PBMCs stimulated with the mitogen phytohemagglutinin in the presence of IL‐4 and IL‐21 fail to secrete detectable IgE, in contrast to cultures treated with IL‐4 alone [172]. Similarly, total B cells isolated from human tonsils and activated with anti‐CD40 plus IL‐4 and IL‐21 exhibited reduced IgE class switching, as measured by IgE^+^ B cell frequency, compared with cultures lacking IL‐21 [167]. These findings align with in vivo mouse studies in which loss of IL‐21 signaling is associated with enhanced IgE responses.

Importantly, the effects of IL‐21 on IgE production in both mouse and human B cell cultures are highly sensitive to culture conditions, including the nature and strength of costimulatory signals, B cell density, and IL‐21 concentration. For example, Caven and colleagues showed that IL‐21 enhanced IgE secretion at low B cell densities but inhibited IgE production at higher densities in mouse and human B cell cultures [173]. The mode of B cell activation is also critical: IL‐21 inhibited IgE production in phytohemagglutinin‐stimulated cultures but enhanced IgE secretion when B cells were activated with anti‐CD40 antibodies [172]. Moreover, the strength of CD40 co‐stimulation modulates IL‐21 activity, as low doses of anti‐CD40 combined with IL‐21 more effectively suppressed IgE class switching than higher doses in both mouse and human B cell cultures [167]. IL‐21 concentration further influences outcomes, with lower doses more potently inhibiting IgE production than higher doses in mouse B cell cultures [168]. Finally, the order of cytokine exposure also matters, as culturing mouse B cells with IL‐4 followed by IL‐21 promoted expansion of IgE^+^ cells without altering IgE class switching [174].

Collectively, these studies highlight substantial variability in IL‐21–mediated regulation of IgE class switching and production in vitro. Rather than indicating contradictory biology, these findings suggest that IL‐21 functions as a context‐dependent modulator of IgE responses, acting in concert with IL‐4 and co‐stimulatory signals. Small changes in the magnitude, timing, or spatial availability of IL‐21, IL‐4, and co‐stimulation can markedly alter IgE outcomes. In vivo, this implies that the balance and spatiotemporal coordination of IL‐4 and IL‐21 production by Tfh cells—and potentially additional signals from follicular dendritic cells or Tfr cells—may fine‐tune humoral immunity to support GC B cell survival and differentiation, coordinating IgE class switching only when appropriate.

### 3.3 IL‐13

Early models proposed a functional dichotomy in which IL‐4 acts as the immunoregulatory type 2 cytokine within SLOs, whereas IL‐13 functions primarily as a tissue effector cytokine [24, 175]. This framework implied a minimal role for IL‐13 in regulating B cell responses in lymphoid tissues. IL‐13 signals through the type II IL‐4 receptor, composed of IL‐4Rα and IL‐13Rα1, leading to STAT6 phosphorylation [176]. Analyzes of IL‐13Rα1 expression identified B cells and monocytes—but not T cells—as IL‐13–responsive populations [177]. Consistent with this, studies largely using human B cells demonstrated that IL‐13 can directly promote B cell survival, proliferation, and IgE class‐switch recombination, albeit generally less potently than IL‐4 [178, 179, 180, 181, 182, 183]. In contrast, early studies in mice suggested that B cells were unresponsive to IL‐13 due to a lack of IL‐13Rα1 expression [184]. However, these studies focused exclusively on naïve B cells, whereas subsequent work showed that IL‐13Rα1 can be induced upon B cell activation [183, 185]. Thus, apparent species differences may instead reflect differences in B cell activation state, as human studies often examined total PBMC‐derived B cells enriched for antigen‐experienced populations, whereas B cells from specific‐pathogen‐free mice are predominantly naïve.

As a strategy to understand allergic sensitization and the regulation of IgE production, our lab has studied hyper‐IgE syndromes, including DOCK8 and STAT3 deficiencies. Using a model of T cell–restricted DOCK8 deficiency (*Cd4* ^*cre*^ *Dock8* ^*fl/fl*^), we observed markedly increased antigen‐specific IgE following LPS and NP‐OVA immunization compared with flox‐only control mice, which had undetectable IgE [9]. In these mice, we identified a distinct subset of Tfh cells that produced high levels of both IL‐4 and IL‐13, which we termed Tfh13 cells. This population was absent in control mice following LPS sensitization. More recently, we found elevated frequencies of circulating Tfh13 cells in the blood of patients with DOCK8 deficiency, suggesting conservation of this population in humans [52]. Importantly, Tfh13 cells are not unique to DOCK8 deficiency: we detect them in wild‐type mice exposed to the aeroallergen *Alternaria*, as well as in peanut and cholera toxin–based food allergy models [9, 52]. Strikingly, mice lacking Tfh13 cells (*Il13* ^*cre*^ *Bcl6* ^*fl/fl*^) or lacking Tfh‐derived IL‐13 (mixed bone marrow chimers using *Cd4* ^*cre*^ *Bcl6* ^*fl/fl*^ and *Il13* ^*−/−*^) exhibit a selective defect in anaphylactic, high‐affinity IgE, while total IgE levels remain largely intact [9]. These findings identify a specific requirement for Tfh‐derived IL‐13 in the generation of high‐affinity IgE and allergic disease. Consistent with this model, we found that GC B cells and plasma cells upregulate IL‐13Rα1, the signaling receptor chain for IL‐13, indicating that IL‐13 may act directly on B cells to promote high‐affinity IgE responses [9].

IL‐13 may also play a role in allergic memory. MBCIIs, which are enriched in allergic individuals, are characterized by high expression of IL‐4Rα and CD23, the low‐affinity IgE receptor, in both humans [58, 59, 60] and mice [60]. Notably, in humans, MBCIIs upregulate *IL13RA1*, suggesting that this reservoir for IgE memory may be responsive to IL‐13 [58, 59, 60]. Supporting the clinical relevance of our findings, we observed increased frequencies of circulating Tfh13 cells in patients with peanut allergy or aeroallergies compared with non‐allergic controls [9]. Interestingly, a recent publication from the Berin lab revealed that Tfh13 cells are present in patients with milk allergy but absent in patients with eosinophilic esophagitis, where Tfh cells instead produce IL‐10 with low levels of IL‐4 [186]. While IL‐4 in eosinophilic esophagitis may permit limited IgE class switching, the absence of IL‐13 likely restricts high‐affinity IgE, consistent with the minimal clinical benefit of anti‐IgE therapy in this disease [186, 187]. In contrast, combined IL‐4 and IL‐13 production by Tfh13 cells in milk allergy supports high‐affinity, pathogenic IgE and immediate hypersensitivity. IgG4 responses in eosinophilic esophagitis, which correlate with disease activity [187, 188], may be driven by IL‐10– and IL‐21–producing CD4^+^ T cell subsets [186]. Interestingly, during helminth infection, both our lab and others have observed an absence of IL‐4/IL‐13–co‐producing Tfh13 cells, with the Tfh compartment instead composed predominantly of IL‐4 single‐producing Tfh cells [9, 19, 24]. Consistent with this, mice infected with the helminth *Nippo* and co‐immunized with NP‐OVA generated very few high‐affinity, NP‐specific IgE antibodies, despite mounting a low‐affinity NP‐specific IgE response [9]. These findings underscore that Tfh13 cells are not a universal feature of type 2 immunity, particularly in settings that do not typically culminate in anaphylaxis. Rather, IL‐4–producing Tfh cells lacking IL‐13 (“Tfh2” cells) appear sufficient to support low‐affinity, antigen‐specific IgE production. Together, these findings reinforce that Tfh cytokines—particularly IL‐13 expression—determine the affinity and pathogenicity of type 2 humoral responses.

Collectively, these data reveal a strong positive association between Tfh13 cells, IL‐13 signaling, and the generation of pathogenic IgE responses. These findings highlight IL‐13 as an attractive therapeutic target in allergy. Consistent with this idea, clinical trials blocking IL‐13 with tralokinumab [189] or lebrikizumab [190] resulted in reduced serum IgE levels in allergic individuals, although these effects may be secondary to inhibition of upstream IL‐13–dependent immune pathways.

### 3.4 IL‐10

Many CD4^+^ T cell subsets are capable of producing IL‐10, including several populations discussed in this review, such as Tregs, Tr1, Tfh, and Tfr cells [50, 87, 106, 107, 191]. IL‐10 is classically viewed as an immunoregulatory cytokine that limits inflammation by suppressing the expression of pro‐inflammatory cytokines, MHC class II, and costimulatory molecules (reviewed by Saraiva et al. [191]). Notably, like IL‐21, IL‐10 signals through STAT3; however, IL‐10–STAT3 signaling preferentially induces an anti‐inflammatory gene expression program, whereas IL‐21–STAT3 signaling is typically associated with inflammatory and effector responses. Beyond its immunoregulatory roles, IL‐10 broadly influences B cell differentiation by synergizing with IL‐4 to enhance human B cell proliferation and IgG secretion [192] and promotes the differentiation of tonsillar GC B cells into plasma cells [193, 194]. Consistent with these findings, disruption of IL‐10 signaling in multiple mouse models impairs GC B cell formation and alters their phenotype [50, 107], while also reducing plasma cell responses [107]. IL‐10 has also emerged as a key cytokine implicated in the regulation of IgE responses.

In vitro studies examining the effects of IL‐10 on IgE class switching and antibody production have yielded divergent results in both mouse and human systems, highlighting strong context dependence. Using human tonsillar B cells, Caven and colleagues reported that IL‐10 enhanced IgE production in culture, either alone or in combination with IL‐21 [173]. In contrast, a separate study using co‐cultures of naïve B cells and Tfr‐like cells isolated from human tonsils found that blockade of IL‐10 receptor signaling resulted in a modest increase in εGLT expression, suggesting that IL‐10 signaling restrains IgE class switching in this setting [106]. Additional complexity emerged from studies using human PBMC cultures. Jeannin and colleagues demonstrated that the timing of IL‐10 exposure critically influenced IgE outcomes: when IL‐10 was added early together with IL‐4, IgE secretion was reduced compared with IL‐4 alone, whereas delayed addition of IL‐10 enhanced IL‐4‐induced IgE production [195]. In both conditions, IgG4 secretion was increased relative to controls. These findings were largely confirmed by a subsequent study in which PBMCs cultured with IL‐4 and IL‐10 showed reduced IgE secretion compared with IL‐4‐only cultures, although IgG4 levels were unaffected in that system [196]. When similar experiments were performed using purified human B cells, Lin and colleagues observed that IL‐10 had no effect on IgE secretion but robustly enhanced IgG4 production. Based on these collective findings, the authors concluded that IL‐10 indirectly modulates IL‐4‐driven IgE class switching and secretion through accessory cells present in PBMC cultures, whereas IL‐10 can act directly on B cells to promote IgG4 production in the absence of additional cellular inputs [196].

Consistent with these variable findings, studies in mouse B cell culture systems have likewise failed to demonstrate a clear role for IL‐10 in promoting or inhibiting IgE class switching, with Yang and colleagues reporting no detectable impact of IL‐10 on IgE CSR in murine B cells [167]. Taken together, these studies indicate that IL‐10 can influence IgE class switching and antibody production under specific experimental conditions, but do not support a model in which IL‐10 functions as a universal promoter or inhibitor of IgE responses.

Similarly, IL‐10 exhibits a complex relationship with IgE responses in vivo. Multiple studies have reported either positive or negative correlations between IL‐10–producing CD4^+^ T cells and serum IgE levels [70, 90, 94]. For example, FOXP3‐deficient mice paradoxically display elevated steady‐state serum IL‐10 compared with wild‐type controls, which positively correlates with increased serum IgE [70]. Likewise, in distinct vaccination models, increased frequencies of IL‐10^+^ CD4^+^ T cells in the lung [94] or elevated IL‐10 secretion from splenocytes [90] were associated with higher serum IgE levels. In contrast, a study by Cañete and colleagues identified an inverse correlation between serum IgE titers and the frequency of IL‐10^+^ CD4^+^ T cells in paired tonsil samples, suggesting that T cell–derived IL‐10—potentially from Tfr‐like cells—may restrain IgE production in humans [106]. Multiple in vivo murine studies have examined the impact of impaired IL‐10 on the generation of IgE antibodies, with mixed results [50, 88, 167]. In an oral tolerance model, one group reported that IL‐10–deficient mice mounted a normal tolerogenic response—characterized by an absence of increased serum IgE—following sensitization with OVA and alum and subsequent intranasal challenge with OVA alone, indicating that IL‐10 was not required to suppress IgE responses in this setting [88]. Similarly, in a protein vaccination model, another study found that IL‐10–deficient mice exhibited frequencies of IgE^+^ GC B cells and antigen‐specific IgE^+^ plasma cells comparable to those of wild‐type mice, again suggesting that IL‐10 is not an essential negative regulator of IgE production in this context [167].

In contrast, other work has demonstrated a positive role for IL‐10 in promoting IgE responses in vivo [50]. As discussed above, using a peanut and cholera toxin food allergy model, Xie and colleagues found that Tfr cells were enriched for *Il10* expression and that B cell–specific deletion of the IL‐10 receptor resulted in impaired GC B cell responses and reduced peanut‐specific IgE production [50]. Moreover, selective impairment of *Il10* expression in Tfr cells through deletion of the transcription factor BLIMP1 in chimeric mice phenocopied the B cell–specific IL‐10 receptor deficiency, leading the authors to conclude that Tfr‐derived IL‐10 promotes antigen‐specific IgE responses in this model [50].

Collectively, these studies highlight the context‐dependent effects of IL‐10 on IgE regulation. Rather than acting as a universal suppressor or enhancer, IL‐10 appears to modulate IgE class switching and antibody production in a manner dependent on the immunization strategy, tissue context, and cellular source, while broadly supporting humoral immunity alongside cytokines such as IL‐4 and IL‐21.

### 3.5 TGF‐β

Like IL‐10, TGF‐β is produced by multiple immune cell subsets, with Tregs serving as a major source that restrains aberrant T cell activation and prevents autoimmunity [197]. In humans, rare mutations in the genes encoding the TGF‐β receptor subunits (*TGFBR1* and *TGFBR2*) are associated with elevated serum IgE and increased susceptibility to allergic diseases, including food allergy and asthma [198, 199], highlighting TGF‐β as a negative regulator of IgE production. In agreement, several human studies demonstrate a negative correlation between TGF‐β expression and allergic symptoms and/or serum IgE levels. For example, PBMCs from children who outgrew milk allergy made more TGF‐β when restimulated with milk proteins in vitro, while PBMCs from milk‐allergic children secreted lower levels of TGF‐β [73]. In another study, it was found that T cells isolated from intestinal biopsies from food allergic children expressed lower levels of TGF‐β relative to healthy controls, which coincided with what appeared to be fewer TGF‐β^+^ cells in the lamina propria of the biopsies from allergic children [200]. Interestingly, single‐nucleotide polymorphisms (SNPs) in the *TGFB1* locus are also associated with increased risk for asthma and elevated serum IgE levels in patients with atopic asthma [201, 202]. However, functional analyzes show that one of these key promoter‐region SNPs actually increases *TGFB1* promoter activity, suggesting a link between increased *TGFB1* expression and elevated IgE levels [203]. These conflicting findings likely reflect the pleiotropic nature of TGF‐β, which acts on multiple cell types and may regulate IgE antibody production both directly and indirectly at several stages of the immune response.

Studies in mice more clearly define TGF‐β as inhibitory to IgE responses, primarily through its role in the induction of tolerance. TGF‐β present in breast milk has been proposed to protect offspring from allergic disease by promoting tolerance to innocuous antigens encountered early in life [204, 205]. Consistent with this, oral administration of TGF‐β together with OVA—either in wild‐type mice or in transgenic mice whose T cells are OVA‐specific—nearly abolished OVA‐specific IgE responses following subsequent OVA or OVA and alum immunization. In contrast, TGF‐β alone was insufficient to suppress IgE production after OVA and alum immunization in wild‐type mice [206]. Conversely, depletion of TGF‐β during oral antigen exposure disrupted tolerance and resulted in robust OVA‐specific IgE responses comparable to those in non‐tolerized mice. Collectively, these findings support a model in which TGF‐β limits IgE responses indirectly by promoting oral tolerance rather than by directly inhibiting IgE induction.

Although exogenous TGF‐β alone is insufficient to suppress IgE responses, multiple studies indicate that intact TGF‐β signaling is required to restrain IgE production at steady state and in allergy models. Similar to humans with TGF‐β receptor mutations, mice heterozygous for *Tgfbr1* exhibit elevated total serum IgE compared with wild‐type controls and spontaneously generate IgE against dietary wheat proteins, consistent with impaired oral tolerance [199]. In a peanut and cholera toxin food allergy model, *Tgfbr1* ^+/−^ mice also develop higher levels of peanut‐specific serum IgE than wild‐type mice [199]. Likewise, mice heterozygous for TGF‐β itself display increased OVA‐specific IgE responses in a murine asthma model, despite only partial reduction of TGF‐β availability [207]. Collectively, these findings demonstrate that TGF‐β signaling is necessary—but not sufficient—to constrain IgE antibody responses, acting primarily to maintain tolerance rather than directly blocking IgE induction.

While TGF‐β can clearly influence IgE production, the underlying mechanisms remain poorly defined. TGF‐β promotes peripheral Treg differentiation and is essential for Tr1 cell development, both of which can dampen inflammation and potentially limit IgE responses; however, these cells may not be appropriately localized within lymph nodes to directly suppress IgE class‐switch recombination. Instead, Tfr cells, which reside within B cell follicles, may be better positioned to directly restrain IgE production, yet whether TGF‐β promotes Tfr differentiation has not been tested.

Another mechanism by which TGF‐β may regulate IgE responses is through modulation of Tfh cell differentiation. The role of TGF‐β in Tfh development remains controversial: in vitro studies suggest that TGF‐β promotes Tfh differentiation of human CD4^+^ T cells [208, 209], whereas murine studies report both positive [210, 211] and negative [209, 212] effects. These discrepancies suggest that TGF‐β may exert more nuanced control over Tfh biology than simply promoting or inhibiting differentiation. Supporting this idea, Haque and colleagues found that patients heterozygous for TGF‐β receptor mutations exhibit increased frequencies of IL‐4^+^ “Tfh2” cells and reduced IL‐17^+^ “Tfh17” cells in circulation compared with healthy controls [199]. This skewing toward IL‐4–producing Tfh subsets provides a potential explanation for the elevated IgE observed in these patients. Collectively, these findings indicate that TGF‐β signaling influences not only Tfh differentiation but also Tfh polarization, thereby indirectly shaping IgE antibody responses.

TGF‐β also acts directly on B cells and is a potent inducer of IgA CSR. In an in vitro screen testing IL‐4 in combination with 15 additional individual cytokines, TGF‐β was the only cytokine that completely inhibited production of εGLTs (IL‐21 was not tested), demonstrating that TGF‐β is sufficient to directly suppress IgE class switching in vitro [133]. Whether this mechanism operates in vivo remains unclear, although Tfr cells are a potential source of TGF‐β [213, 214].

Overall, TGF‐β is a multifaceted cytokine with complex in vivo functions that likely inhibit CSR and antibody production through both direct and indirect mechanisms. Although TGF‐β dampens IgE responses and has important implications for oral tolerance, asthma, and atopy, it may also contribute pathogenically to obstructive airway diseases [215]. Thus, tight regulation of TGF‐β signaling is essential to balance its anti‐inflammatory and profibrotic effects in allergic disease, likely limiting its therapeutic potential in allergy.

## 4 Early Allergen Exposure Shapes the T Cell Landscape and IgE Responses

The experimental models discussed above highlight how distinct CD4^+^ T cell subsets and their associated cytokines—particularly IL‐4–producing Tfh2 cells and IL‐4/IL‐13–co‐producing Tfh13 cells—shape the magnitude, affinity, and pathogenic potential of IgE responses. These findings raise an important question: do similar T cell–dependent mechanisms govern the evolution of allergen‐specific humoral immunity in humans? Longitudinal studies of early‐life allergen exposure provide a unique opportunity to address this question, revealing how regulatory versus pathogenic T cell programs influence isotype selection, epitope specificity, and the risk of clinical allergy.

**FIGURE 1:**
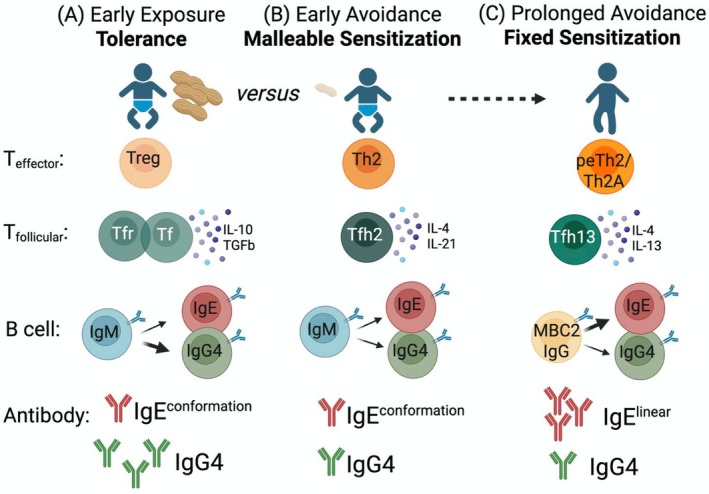
Proposed model of T and B cell characteristics in tolerance versus sensitization to food allergens. Immune features of tolerance during early life (less than 1 years of age) with food allergen consumption (A), or metastable sensitization during that same period in infants with peanut avoidance (B). This progresses to established allergy after prolonged avoidance in young children (C). The state shown in B with early sensitization is proposed to be reversible in that T and B cell populations might not exist in long‐lived states and could potentially be suppressed with interventions.

The LEAP study fundamentally reshaped our understanding of early‐life immune programming to food antigens. Early introduction of peanut dramatically reduced the risk of peanut allergy and led to sustained clinical tolerance [216, 217, 218]. Viewed alongside foundational work on mechanisms of tolerance versus sensitization, these findings suggest that early allergen exposure imprints durable, antigen‐specific T cell and B cell states that bias the immune system away from pathogenic IgE responses and toward long‐term tolerance. Immunologically, a protective state has been associated with the induction of regulatory T cell populations, including Tregs and Tfr cells, as well as an IgG4‐biased B cell response in humans [73, 74, 77, 78, 85, 106] (Figure 1A). In contrast, avoidance of peanut was associated with a significantly higher incidence of peanut allergy [218, 219] (Figure 1B). Importantly, peanut‐specific IgE could be detected in both consumption and avoidance groups, albeit with differences in magnitude and epitope specificity. Over time, children who avoided peanut preferentially developed IgE recognizing linear epitopes of major peanut allergens (Ara h 1, 2, and 3), a feature strongly associated with symptomatic food allergy [220, 221]. In contrast, children who consumed peanut generated IgE that preferentially bound conformational epitopes, alongside IgG4 directed against linear epitopes [220]. These findings suggest that allergen avoidance permits progressive diversification and maturation of the B cell response across broader regions of the allergen, potentially increasing IgE affinity and pathogenicity (Figure 1C). Thus, allergen‐specific IgE responses evolve over the first years of life in susceptible individuals.

In parallel with Tfh‐ and Tfr‐regulated humoral responses, effector T cell populations are detectable in the peripheral blood of peanut‐allergic individuals, including pathogenic effector Th2 (peTh2) [63, 85, 222] or Th2A cells [64]. These cells may amplify tissue inflammation, instruct innate effector populations, and potentially contribute to local class‐switching events within tissues. We propose that with continued allergen avoidance, the emergence or stabilization of a Tfh13 population promotes sequential class switching of high‐affinity, post–germinal center IgG1^+^ MBCII cells to high‐affinity IgE (Figure 1C), a state associated with increased risk of symptomatic allergy and anaphylaxis [9, 26, 58, 59, 60, 186, 218]. In contrast, regular consumption appears to favor an isotype profile dominated by IgG subclasses, potentially driven by regulatory T cell populations—including Tregs and Tfr cells—through mechanisms that may involve TGF‐β, although the precise molecular pathways remain incompletely defined. Such IgG responses are associated with epitope masking and inhibitory Fc receptor engagement, thereby limiting mast cell and basophil activation. Interestingly, despite the well‐established role of TGF‐β in promoting IgA class switching, IgA may not be a dominant correlate of this more tolerogenic immune state [223, 224].

In summary, IgE responses do not arise from a singular “type 2” pathway but instead reflect the integrated actions of multiple T cell subsets operating across tissues and SLOs. From early cytokine conditioning by iNKT cells and dendritic cells to the specialization of Tfh2 and Tfh13 cells within germinal centers, to the counterbalancing effects of Tregs and Tfr cells, the quality, location, and timing of T cell help determine whether IgE responses remain low‐affinity and clinically silent or evolve into high‐affinity, pathogenic antibodies capable of driving anaphylaxis. Human studies, including early‐life allergen exposure paradigms, reinforce the concept that it is not merely the presence of IgE but the nature of T cell help—regulatory versus pathogenic, IL‐13–competent versus IL‐13–restricted—that shapes disease risk. A deeper understanding of how these T cell programs are initiated, maintained, and interconverted will be essential for developing therapies that selectively disrupt pathogenic IgE while preserving protective immunity.

## Funding

This work was supported by the Food Allergy Science Initiative (FASI—S.C.E.), The Food Allergy Fund (FAF—S.C.E. and A. W.), R01 AI136942 (S.C.E. and A. W.), R01 AI168016 (S.C.E and A.W), T32 AI007476 (A.L.T).

## Conflicts of Interest

The authors declare no conflicts of interest.

## Data Availability

Data sharing not applicable to this article as no datasets were generated or analyzed during the current study.
